# The demanding attention of tuberculosis in allogeneic hematopoietic stem cell transplantation recipients: High incidence compared with general population

**DOI:** 10.1371/journal.pone.0173250

**Published:** 2017-03-09

**Authors:** Hyo-Jin Lee, Dong-Gun Lee, Su-Mi Choi, Sun Hee Park, Sung-Yeon Cho, Jae-Ki Choi, Si-Hyun Kim, Jung-Hyun Choi, Jin-Hong Yoo, Byung-Sik Cho, Ki-Seong Eom, Seok Lee, Yoo-Jin Kim, Hee-Je Kim, Chang-Ki Min, Dong-Wook Kim, Jong-Wook Lee, Woo-Sung Min, Jung Im Jung

**Affiliations:** 1 Division of Infectious Diseases, Department of Internal Medicine, College of Medicine, The Catholic University of Korea, Seoul, Republic of Korea; 2 Vaccine Bio Research Institute, College of Medicine, The Catholic University of Korea, Seoul, Republic of Korea; 3 The Catholic Blood and Marrow Transplantation Center, College of Medicine, The Catholic University of Korea, Seoul, Republic of Korea; 4 Department of Radiology, College of Medicine, The Catholic University of Korea, Seoul, Republic of Korea; University of Sassari, ITALY

## Abstract

**Background:**

The risk of developing tuberculosis (TB) in allogeneic hematopoietic stem cell transplantation (HSCT) recipients is expected to be relatively high in an intermediate TB burden country. This single-center retrospective study was conducted to investigate risk factors and the incidence of TB after allogeneic HSCT.

**Methods:**

From January 2004 to March 2011, 845 adult patients were enrolled. Starting April 2009, patients were given isoniazid (INH) prophylaxis based on interferon-γ release assay results. The incidence of TB was analyzed before and after April 2009, and compared it with that of the general population in Korea.

**Results:**

TB was diagnosed in 21 (2.49%) of the 845 allogeneic HSCT patients. The median time to the development of TB was 386 days after transplantation (range, 49–886). Compared with the general population, the standardized incidence ratio of TB was 9.10 (95% CI; 5.59–14.79). Extensive chronic graft-versus-host disease (GVHD) was associated with the development of TB (*P* = 0.003). Acute GVHD, conditioning regimen with total body irradiation and conditioning intensity were not significantly related. INH prophylaxis did not reduce the incidence of TB (*P* = 0.548). Among 21 TB patients, one patient had INH prophylaxis.

**Conclusion:**

Allogeneic HSCT recipients especially those who suffer from extensive chronic GVHD are at a high risk of developing TB. INH prophylaxis did not statistically change the incidence of TB, however, further well-designed prospective studies are needed.

## Introduction

*Mycobacterium tuberculosis* is an acid-fast, aerobic bacillus, which is one of the leading infectious diseases around the world [1, 2]. Tuberculosis (TB) is also well known for a significant opportunistic infection among hematopoietic stem cell transplantation (HSCT) recipients, although it is 10 times less frequent than in solid organ transplant patients [2, 3]. The immune system of HSCT recipients are suppressed as a result of their hematologic diseases, chemotherapy, radiation, immunosuppressive therapy, and graft-versus-host disease (GVHD) [1]. Thus, the incidence of TB among allogeneic HSCT recipients is reportedly 2–40 times higher than that of the general population [1, 4–6]. In particular, diagnosis of TB is frequent in Korea, with an estimated TB incidence rate of 97 cases per 100 000 persons in the general population in 2013 according to World Health Organization report [7].

Isoniazid (INH) prophylaxis in patients with latent TB infection (LTBI) is recommended for the prevention of active TB disease [2]. However, INH prophylaxis guidelines have not yet been adopted by many centers in Korea. There are a few studies on TB and INH prophylaxis in HSCT recipients.

The purpose of this study was to examine the incidence of TB disease after allogeneic HSCT as compared with the general population, identify risk factors for TB disease, and analyze the efficacy of INH prophylaxis.

## Materials and methods

### Study design and subjects

We retrospectively reviewed the medical records of all consecutive adult patients (age ≥18 years) who underwent allogeneic HSCT for acute myeloid leukemia (AML), acute lymphoblastic leukemia (ALL), chronic myeloid leukemia (CML), multiple myeloma (MM), or myelodysplastic syndrome (MDS), from January 2004 to March 2011, at the Catholic Blood and Marrow Transplantation Center, Seoul, Korea. Patients undergoing a second transplantation, or with human immunodeficiency virus infection were excluded from the study. Those who were taking anti-TB medication before or during the HSCT were excluded as well. The demographic features, laboratory and radiologic data of study participants were carefully examined (For details see S1 Data). The end of this study was set at December 2014 or time of death or the date of loss to follow-up.

As of April 2009, patients who planned to undergo allogeneic HSCT were given INH prophylaxis (300 mg/day) based on interferon-γ release assay (IGRA) results. Korea Centers for Disease Control and Prevention has recommended INH prophylaxis for latent TB since 2008 [8, 9]. In accordance with the national policy for latent TB, the Catholic Blood and Marrow Transplantation Center in Korea changed guidelines for TB prevention in April 2009. INH prophylaxis was started on the day that the IGRA results were made available, and continued for 9 months after HSCT. The IGRA results were evaluated using QuantiFERON^®^-TB Gold In-Tube (QFT-GIT; Cellestis Ltd., Victoria, Australia). The incidence of TB in each study group was analyzed (group A, allogeneic HSCT between January 2004 and March 2009, n = 550; group B, allogeneic HSCT between April 2009 and March 2011, n = 295).

This study obtained approval from the Institutional Review Board of Seoul and Yeouido St. Mary’s Hospital with a waiver of informed consent. (No. KC15RIMI0162, SC10RISI0023)

### Definitions

#### Definitions of TB and assessment of treatment outcome

The diagnosis of TB was considered proven if *M*.*tuberculosis* was cultured from any of the clinical specimens. The diagnosis of a probable case of active TB required at least one of the following criteria in patients with clinical symptoms and signs suggestive of TB disease: 1) acid-fast bacilli from clinical specimen (sputum, body fluid, *etc*.) present; 2) tissue histology suggestive of TB such as caseous necrosis with or without granuloma or positive Ziehl-Neelsen staining; or 3) fluid cytology (pleural or pericardial effusion or ascites or cerebrospinal fluid [CSF]) showing exudates with lymphocyte predominance and high adenosine deaminase (>40 IU/L in the body fluid, or >10 IU/L in case of CSF), when the case had no evidence of malignancy. A possible case was defined when a patient with clinical symptoms and signs, had radiological findings suggestive of TB at the time of diagnosis without definite evidence of another infectious cause, and the patient’s condition improved with anti-TB treatment when other antibacterial and antifungal agents were ineffective [6, 10–16]. Miliary TB was defined as described in a previous study [17]. The radiological findings were confirmed by two highly-experienced radiologists. Polymerase chain reaction for TB was used only to distinguish TB and non-tuberculous mycobacterial infection, not for TB diagnosis. The IGRA results had no influence on the diagnosis of TB. Clinical response of TB treatment was classified as cure, improvement, or failure [16]. The definition of Immune reconstitution inflammatory syndrome was given as previously [18].

#### Transplantation procedure and diagnosis of GVHD

All patients received oral ciprofloxacin (250–500 mg twice a day) as antibacterial prophylaxis. Itraconazole oral solution (2.5 mg/kg twice a day) or micafungin (50 mg daily) were used as primary antifungal prophylaxis, from the first day of conditioning until engraftment. Fluconazole (400 mg daily) prophylaxis after engraftment was administered according to GVHD status and the national guideline [19]. Trimethoprim-sulfamethoxazole (one single-strength tablet daily) was administered as prophylaxis for *Pneumocystis jirovecii* infection after engraftment until discontinuation of the immunosuppressants. The patients also received a herpes simplex virus prophylaxis of acyclovir (400–800 mg orally twice a day or 5 mg/kg intravenously two or three times a day) from day 7 to engraftment for transplants of matched related donors. Patients who underwent allogeneic HSCT from mismatched related donors, unrelated donors, or umbilical cord blood received a higher dose of intravenous acyclovir (10 mg/kg three times a day) until engraftment. To prevent cytomegalovirus disease after engraftment, the preemptive strategy was conducted in a CMV DNA load-guided, risk-adapted manner [13]. The myeloablative conditioning regimen was used when at least one of the following criteria was met: 1) total body irradiation ≥ 500 cGy as a single fraction or ≥ 800 cGy as fractionated; 2) busulfan 4 mg kg/day administered for 4 days, combined with cyclophosphamide 120 mg/kg; 3) total busulfan of ≥ 9 mg/kg; and 4) melphalan of ≥ 140 mg/m^2^. The reduced intensity conditioning regimen was used when meeting any of the following criteria: 1) total body irradiation < 500 cGy as a single fraction or < 800 cGy as fractionated; 2) total busulfan of < 9 mg/kg; 3) melphalan of < 140 mg/m^2^; 4) thiotepa of < 10 mg/kg; and 5) the carmustine, etoposide, cytarabine, and melphalan regimen [20]. Diagnoses and grades of acute GVHD were made based on the consensus criteria [21]. The definition of chronic GVHD was given by using the revised Seattle classification [20]. The patients concomitant treated with cyclosporine and rifampicin were frequently monitored for therapeutic drug levels of cyclosporine.

### Statistical analysis

Overall survival (OS), disease-free survival (DFS), and the cumulative incidences of TB disease were considered the main end points of this study. OS and DFS results were plotted using the Kaplan-Meier method and compared using log-rank test. The cumulative incidences of TB disease under which method non-TB disease was used as a competing risk event of TB disease, were plotted and compared using the Gray’s test. To evaluate the potential factors for TB disease, categorical variables were analyzed using a χ^2^ or Fisher’s exact test. For multivariate analysis, variables with *P*-value of < 0.30, on univariate analysis were entered into the model selection procedure on the basis of Cox proportional hazard modeling.

Two-sided *P* values < 0.05 were considered statistically significant. The standardized incidence ratios (SIR) of TB disease were calculated to compare the incidence of TB disease in our cohort with that of the general population in Korea, whose data were collected from the annual reports of the Korea Centers for Disease Control and Prevention between 2004 to 2011 [22]. SIR with corresponding 95% confidence intervals (CI) were calculated using Poisson regression. Statistical studies were performed with the Statistical Package for the Social Sciences version 13.0 (SPSS, Inc., Chicago, IL, USA) and the cumulative incidence analyses were performed using with R (freely distributed on the web, http://cran.r-project.org/).

## Results

### Overall HSCT recipient characteristics and risk factors for the development of TB

**Fig 1:**
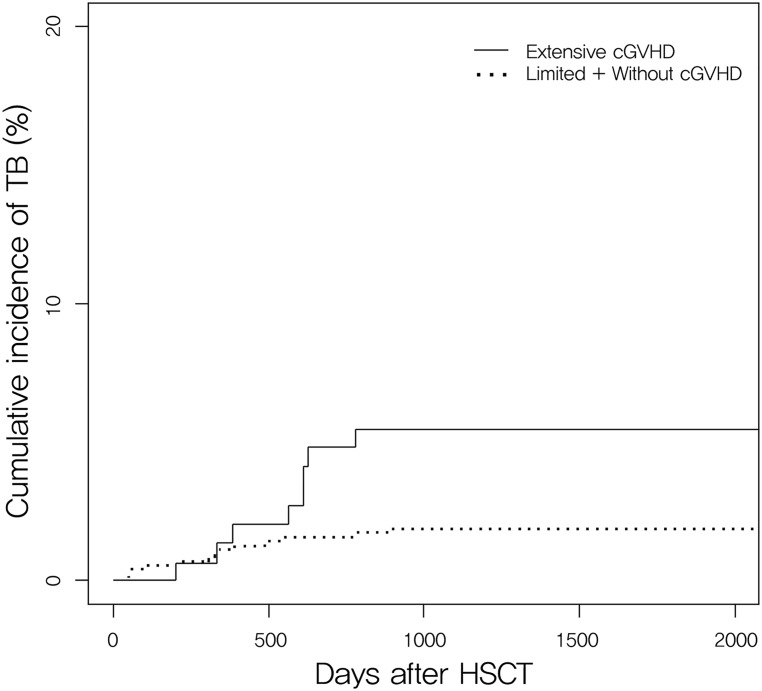
Cumulative incidence of tuberculosis in allogeneic hematopoietic stem cell transplantation recipients with and without chronic graft-versus-host-disease. Extensive chronic GVHD was associated with the development of TB (*P* = 0.003) There is a 4.89 ± 1.32% probability of having TB disease at 1000 days after allogeneic HSCT for extensive chronic GVHD and 1.42 ± 0.50% for limited or without chronic GVHD. cGVHD, chronic graft-versus-host disease; HSCT, hematopoietic stem cell transplantation; TB, tuberculosis.

From January 2004 to March 2011, a total of 861 patients with acute leukemia, CML, MDS, and MM underwent allogeneic HSCT. During the study period, there were 9 cases of second transplantation and 7 patients were taking anti-TB medication prior to the HSCT. After these patients were excluded, a total of 845 patients were enrolled in this study. None of the patients was human immunodeficiency virus-positive. Table 1 shows the general characteristics of the transplantations and the clinical information of the study population. Post-HSCT TB occurred in 21 (2.49%) of the 845 patients in the study. The median follow-up duration of the survivors was 2,044 days (range, 108–3,916). On multivariate analysis (Table 2), risk factors for post-HSCT TB revealed that extensive chronic GVHD was only associated with TB in the study population (*P* = 0.008). Acute GVHD, conditioning regimen with total body irradiation, and conditioning intensity were not significantly related. According to Gray’s test (Fig 1), there was a 4.89 ± 1.32% probability of having TB disease 1000 days after allogeneic HSCT for extensive chronic GVHD and 1.42 ± 0.50% for limited or without chronic GVHD (*P* = 0.003). There was no significant influence of chronic GVHD site on TB development by logistic regression. Chronic GVHD occurred in the skin (TB patients vs. Non-TB patients; 47.6% vs. 40.7%, *P* = 0.523), in the mouth (TB patients vs. Non-TB patients; 19.0% vs. 30.5%, *P* = 0.268), in the liver (TB patients vs. Non-TB patients; 33.3% vs. 19.8%, *P* = 0.134), in the eye (TB patients vs. Non-TB patients; 14.3% vs. 12.5%, *P* = 0.807), in the lung (TB patients vs. Non-TB patients; 19.0% vs. 9.7%, *P* = 0.168), in the gastro-intestinal tract (TB patients vs. Non-TB patients; 14.3% vs. 5.8%, *P* = 0.122) in that order. The outcome of chronic GVHD treatment showed also no association with TB development (*P* = 0.206). Grade of chronic GVHD was associated with TB development (*P* = 0.006). Severe chronic GVHD was 19.0% in TB patients and 13.8% in non-TB patients while moderate chronic GVHD was 47.6% and 18.1%, and mild chronic GVHD was 14.3% and 17.2%, respectively.

**Table 1:** Overall characteristics of allogeneic hematopoietic stem cell transplantation recipients with or without tuberculosis.

Characteristics	TB (n = 21)	No TB (n = 824)	*P*
Age (median, range)	41 (18–60)	40 (18–72)	0.960
Sex (M,%)	10 (47.6)	473 (57.4)	0.371
Transplantation year (%)			0.280
• Jan. 2004-Mar. 2009	16 (76.2)	534 (64.8)	
• Apr. 2009-Mar. 2011	5 (23.8)	290 (35.2)	
Diagnosis (%)			0.569
• AML	12 (57.1)	399 (48.4)	
• ALL	4 (19.4)	227 (27.5)	
• MDS	4 (19.4)	118 (14.3)	
• CML	0 (0)	53 (6.4)	
• MM	1 (4.8)	27 (3.3)	
Donor type (%)			0.870
• Matched sibling	12 (57.1)	456 (55.3)	
• Alternative^a^	9 (42.9)	368 (44.7)	
Stem cell source (%)			0.163
• BM	11 (52.4)	388 (47.1)	
• PB	8 (38.1)	406 (49.3)	
• BM+PB	2 (9.5)	19 (2.3)	
• Cord blood	0 (0)	11 (1.3)	
Conditioning regimen (%)			0.918
• TBI based	15 (71.4)	597 (72.5)	
• no-TBI based	6 (28.6)	227 (27.5)	
ATG given as conditioning (%)			0.662
• Yes	5 (23.8)	232 (28.2)	
• No	16 (76.2)	592 (71.8)	
Conditioning intensity (%)			0.779
• MAC	13 (61.9)	485 (58.9)	
• RIC	8 (38.1)	339 (41.1)	
GVHD prophylaxis (%)			0.693
• CS based	12 (57.1)	435 (52.8)	
• FK based	9 (42.9)	389 (47.2)	
Acute GVHD (%)			0.983
• Grade 0-I	13 (61.9)	512 (62.1)	
• Grade II-IV	8 (38.1)	312 (37.9)	
Chronic GVHD (%)			0.003
• None + Limited	8 (38.1)	569 (69.1)	
• Extensive	13 (61.9)	255 (30.9)	
Relapse			0.597
• Yes	4 (19.0)	198 (24.0)	
• No	17 (81.0)	626 (76.0)	
Follow-up duration, median days (range)			0.268
• Patients who died	343 (121–799)	259 (2-2959)	
• Patients alive at last follow-up	2353 (258–3217)	2037 (108–3916)	

AML, acute myeloid leukemia; ALL, acute lymphoblastic leukemia; ATG, anti-thymocyte globulin; BM, bone marrow stem cell; CML, chronic myelogenous leukemia; CS, cyclosporine; FK, tacrolimus; GVHD, graft-versus-host disease; MAC, myeloablative conditioning; MDS, myelodysplastic syndrome; MM, multiple myeloma; PB, peripheral blood stem cell; RIC, reduced intensity conditioning; TB, tuberculosis; TBI, total body irradiation.

^a^ alternative donor included unrelated donors and mismatched relatives.

**Table 2:** Risk factors for tuberculosis in patients after allogeneic hematopoietic stem cell transplantation.

Variables	Univariate analysis		Multivariate analysis	
	HR (95% CI)	*P*	HR (95% CI)	*P*
Age >40 years	1.29 (0.55–3.03)	0.565		
Male sex	0.72 (0.31–1.70)	0.452		
Transplantation year (%)				
• Jan. 2004-Mar. 2009	1		1	
• Apr. 2009-Mar. 2011	0.57 (0.21–1.57)	0.278	0.55 (0.20–1.51)	0.242
Donor type				
• Matched sibling	1			
• Alternative	1.00 (0.42–2.38)	0.997		
Stem cell source				
• BM	1		1	
• PB	0.72 (0.29–1.80)	0.485	0.62 (0.24–1.57)	0.312
• Others	2.46 (0.55–11.09)	0.242	2.45 (0.54–11.06)	0.244
TBI based Conditioning regimen	0.92 (0.36–2.36)	0.857		
ATG given as conditioning	0.88 (0.32–2.40)	0.798		
Conditioning intensity				
• MAC	1			
• RIC	1.13 (0.47–2.72)	0.792		
GVHD prophylaxis				
• CS based	1			
• FK based	1.15 (0.75–2.73)	0.753		
Acute GVHD (grade ≥ II)	1.01 (0.42–2.43)	0.989		
Extensive chronic GVHD	2.84 (1.18–6.86)	0.020	3.38 (1.37–8.32)	0.008

All variables with values of *P* < 0.300 on univariate analysis were included in the multivariate analysis on the basis of Cox proportional hazard modeling.

ATG, anti-thymocyte globulin; BM, bone marrow stem cell; CI, confidence interval; CS, cyclosporine; FK, tacrolimus; GVHD, graft-versus-host disease; HR, hazard ratio; MAC, myeloablative conditioning; PB, peripheral blood stem cell; RIC, reduced intensity conditioning; TBI, total body irradiation.

### Clinical characteristics of patients with TB

Of the 21 patients with TB after allogeneic HSCT, 10 patients (47.6%) had a previous history of active TB prior to allogeneic HSCT. There were 9 proven, 6 probable, and 6 possible cases in TB diagnosis. Median time from transplantation to TB diagnosis was 386 days (range, 49–886). Eleven of the 21 cases were pulmonary TB, while the other 10 were extrapulmonary TB (47.6%). There were two patients with miliary TB. Median time from onset of symptoms and signs to TB diagnosis was 23 days (range, 0–126). No differences in the outcome were observed for the cases diagnosed with tuberculosis before or after 100 days of HSCT.

17 patients (81.0%) received first-line anti-TB treatment (INH, rifampicin, ethambutol, and pyrazinamide) and 4 patients received second-line therapy. Among 9 cases of proven TB, 6 were subjected to an antimicrobial susceptibility test. Five of these cases had susceptible results to anti-TB medication, while only 1 case had INH resistance. The reasons for the administration of second-line therapy were drug resistance (patient No. 6), first line treatment failure (patient No. 18), and drug intolerance (patient No. 7 and 14). The median time of anti-TB treatment duration was 12 months (range, 1–18 months). Two patients (9.5%) showed anti-TB treatment failure. Patient No. 8 died of variceal bleeding within the esophagus due to liver cirrhosis and didn’t show any improvement despite acid-fast bacilli cultures all being susceptible to anti-TB medications. Patient No. 14 died of pneumonia. This patient was administered INH, ethambutol, pyrazinamide, and levofloxacin. Another 5 patients died for various reasons despite successful anti-TB treatment. Three patients (No 4, 8, and 15) developed immune reconstitution inflammatory syndrome. There wasn’t any case of relapse after anti-TB treatment administered during the study period. The clinical characteristics and diagnostic and treatment data of the patients with TB after HSCT are summarized in Table 3.

**Table 3:** Clinical characteristics of patients with tuberculosis after allogeneic hematopoietic stem cell transplantation.

Patient No.	Age/Sex	Dx	The year of HSCT	Acute GVHD, grade	Chronic GVHD (site, grade)	Therapeutic approach of chronic GVHD	Outcome of chronic GVHD	Relapse of hematologic disease, Y/N	Previous history of TB	Category of TB	Time to Dx of TB after HSCT, day	Site of infection	Treatment	Treatment duration, month	Treatment outcome
1	20/F	AML	2004	0	None			N	N	Possible	311	Lung	HERZ^a^	2	Improved
2	40/M	AML	2004	0	Extensive (mouth, liver, severe)	Steroid pulse therapy, cyclosporine, and tacrolimus	Aggravation	N	Y	Possible	536	Spine	HERZ^a^	18	Cure
3	38/F	AML	2004	II	Extensive (BOOP, liver, skin, GI, moderate)	Steroid pulse therapy	Aggravation	N	Y	Possible	207	Lung	HERZ^a^	4	Improved
4	33/M	AML	2004	III	Extensive (skin, moderate)	Tacrolimus	Improvement	N	Y	Probable	612	Pleura	HERZ^a^	12	Cure
5	18/M	ALL	2004	II	Limited (liver, moderate)	Steroid and cyclosporine	Improvement	Y	N	Probable	491	Lymph node	HERZ^a^	12	Cure
6	52/F	AML	2005	0	None			N	N	Proven	50	Lymph node	2^nd^ line^b^	13	Improved
7	39/M	AML	2005	0	Extensive (liver, moderate)	Steroid pulse therapy and tacrolimus	Improvement	N	N	Probable	328	Lung (miliary)	2^nd^ line^c^	12	Cure
8	50/M	MDS	2005	0	None			N	Y	Proven	217	Lung (miliary)	HERZ^a^	1	Failed
9	43/M	AML	2005	0	Extensive (mouth, skin, 10liver, severe)	Steroid and tacrolimus	Improvement	N	Y	Proven	340	Lung	HERZ^a^	14	Cure
10	41/M	AML	2006	0	Limited (skin, mild)	Tacrolimus	Improvement	N	Y	Possible	49	Lung	HERZ^a^	10	Improved
11	44/F	MM	2006	0	Extensive (mouth, skin, moderate)	Steroid and tacrolimus	Improvement	Y	Y	Possible	57	Lymph node	HERZ^a^	10	Improved
12	24/F	AML	2006	II	Extensive (skin, liver, moderate)	Steroid and tacrolimus	Improvement	N	N	Proven	631	Lung	HERZ^a^	12	Cure
13	34/F	ALL	2007	0	Extensive (mouth, GI, severe)	Steroid and mycophenolate Mofetil	Improvement	N	N	Probable	568	Pericardium, Pleura	HERZ^a^	12	Improved
14	60/F	ALL	2007	0	None			N	N	Proven	93	Pleura	2^nd^ line^c^	1	Failed
15	29/M	ALL	2008	II	Extensive (GI, moderate)	Steroid and tacrolimus	Improvement	N	N	Proven	386	Lung	HERZ^a^	12	Cure
16	40/M	AML	2008	III	Extensive (skin, BO, severe)	Steroid, tacrolimus, and mycophenolate Mofetil	Improvement	N	N	Probable	331	Pericardium	HERZ^a^	10	Improved
17	50/F	MDS	2009	0	Extensive (BOOP, moderate)	Steroid, tacrolimus, and mycophenolate Mofetil	Improvement	Y	N	Proven	393	Lung	HERZ^a^	8	Cure
18	59/F	AML	2009	II	Extensive (eye, skin, liver, moderate)	Cyclosporine	Aggravation	N	Y	Proven	617	Lung	2^nd^ line^d^	17	Cure
19	55/F	MDS	2009	0	Limited (eye, mild)	Steroid and cyclosporine	Improvement	N	Y	Possible	770	Spine	HERZ^a^	12	Cure
20	55/M	AML	2010	II	Extensive (skin, eye, BO, moderate)	Steroid, tacrolimus, and mycophenolate Mofetil	Improvement	N	Y	Proven	782	Lung	HERZ^a^	9	Improved
21	52/F	AML	2010	0	Limited (skin, mild)	Tacrolimus	Improvement	N	N	Probable	886	Lymph node	HERZ^a^	12	Cure

AML, acute myeloid leukemia; ALL, acute lymphoblastic leukemia; BO, bronchiolitis obliterans; BOOP, bronchiolitis obliterans organizing pneumonia; Dx, diagnosis; GI, gastrointestinal; GVHD, graft-versus-host disease; HERZ, treatment regimen of isoniazid, ethambutol, rifampicin, pyrazinamide; HSCT, hematopoietic stem cell transplantation; MDS, myelodysplastic syndrome; MM, multiple myeloma; TB, tuberculosis.

^a^ HERZ was replaced by HER after 2 months.

^b^ 2^nd^ line therapy included ethambutol, cycloserine, levofloxacin, and prothionamide

^c^ 2^nd^ line therapy included levofloxacin, isoniazid, and ethambutol.

^d^ 2^nd^ line therapy included levofloxacin, p-aminosalicylic acid, pyrazinamide, and streptomycin.

### Incidence ratio of TB in HSCT recipients compared with general population

The incidence ratio of post-HSCT TB compared with the general population is shown in Table 4. The overall incidence of TB in allogeneic HSCT recipients was significantly higher than that in the general population in Korea (SIR, 9.10, 95% CI, 5.59–14.79; *P* < 0.001). The incidence of TB in allogeneic HSCT recipients was 654.2 per 100 000 patients per year. There was no statistically significant difference in post-HSCT TB incidence ratio before and after applying INH prophylaxis practice. (*P* = 0.280).

**Table 4:** The incidence of tuberculosis in allogeneic hematopoietic stem cell transplantation recipients compared with the general population in Korea.

Transplant period^a^	Duration of observation, patient-years	TB patients		SIR	95% CI	*P*
		Observed *n* (%)	Expected *n*			
Total (n = 845)	3210.26	21 (2.49)	2.30	9.10	5.59–14.79	< 0.001
Jan. 2004-Mar. 2009 (n = 550)	2341.64	16 (2.91)	1.65	9.58	5.57–16.48	< 0.001
Apr. 2009-Mar. 2011 (n = 295)	868.62	5 (1.69)	0.64	7.73	3.13–19.13	< 0.001

TB, tuberculosis; SIR, standardized incidence ratio; CI, confidence interval.

^a^ Starting April 2009, patients were given isoniazid prophylaxis based on interferon-γ release assay results.

### INH prophylaxis in latent TB

**Fig 2:**
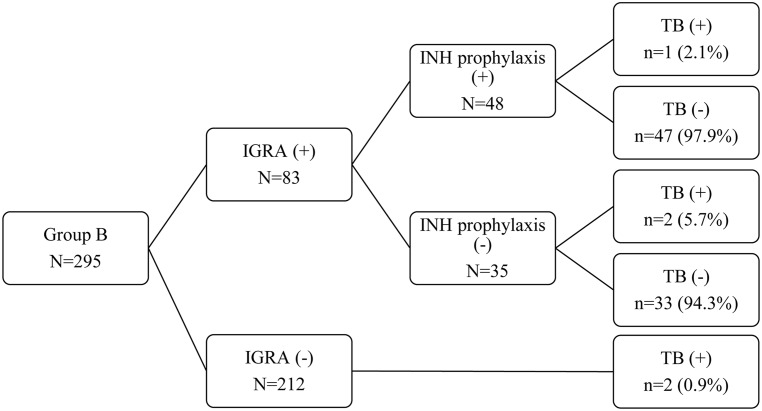
Isoniazid prophylaxis and tuberculosis. INH prophylaxis did not significantly reduce the incidence of TB (*P* = 0.548). Among 5 TB patients in group B, one patient had INH prophylaxis. IGRA negative included indeterminate results. IGRA, interferon-γ release assays; INH, isoniazid; TB, tuberculosis.

Fig 2 shows the rates of INH prophylaxis use and TB disease in group B (allogeneic HSCT between April 2009 and March 2011). Among the 83 IGRA positive patients, 35 patients (42%) were not administered INH prophylaxis or had to stop the prophylaxis. Twenty-seven patients did not start INH prophylaxis due to poor compliance among doctors (8 cases), GVHD (8), hepatotoxicity (8), poor compliance among recipients (2), and esophageal candidiasis (1). Eight patients had to stop INH prophylaxis due to hepatotoxicity (2 cases), GVHD (1), peripheral neuropathy (1), drug eruption (1), drug-induced diarrhea (1), relapses of underlying diseases (1), and poor compliance among recipients (1). The reasons for poor compliance among doctors were the regulation of clinical studies, missed orders, or the ignorance of results. Five patients had TB disease, and 2 patients had an IGRA negative result and underwent allogeneic HSCT without INH prophylaxis. Another 2 patients had IGRA positive results, but they did not take INH prophylaxis. One patient taking INH prophylaxis had TB; however, the INH was taken for only 3 months due to poor compliance. IGRA result (*P* = 0.341) and INH prophylaxis (*P* = 0.548) did not significantly alter the incidence of TB.

## Discussion

The objective of this study was to investigate the incidence of TB in allogeneic HSCT recipients compared with that in the general population in Korea. We also examined the risk factors for the development of TB and the efficacy of INH prophylaxis.

The TB incidence of HSCT recipients is affected by geographical prevalence. Studies on TB development after HSCT published from 1990 to January 2015 are summarized in Table 5. The annual TB incidence of the general population in Korea is about 70–100 cases per 100 000 patients [7, 13]. In our study, active TB after allogeneic HSCT occurred in 2.49% of patients, which is higher than that reported in many western countries, even though study periods and cohorts varied. Taiwan, India, and Australia showed a similar TB incidence (2.3–3.1%). However, Hong Kong and Pakistan had higher incidences than that reported here. Previous studies of TB disease after allogeneic HSCT in Korea demonstrated a diverse range of incidences of 3.3–4.5% (Table 5).

**Table 5:** Description of studies of tuberculosis after hematopoietic stem cell transplantation published from 1990 to January 2015.

Authors/ publishing year	Country (study period)	TB/ No. of HSCT	TB incidence after HSCT	INH prophylaxis	TB of general population	Onset of TB from HSCT (range)	Pulmonary/ Extrapulmonary TB	TB related death
Fan *et al*./2015[1]	Taiwan (1997–2006)	32/1368(Allo) 7/672 (Auto)	2.3% (Allo) 1.0% (Auto)	ND	0.38%	0.06 to 4.63 y	34/5	ND
Sester *et al*./2014[23]	Europe (Jun. 2008-May. 2011)	0/103	0%	18 (17.5%)	0%^a^	ND	ND	ND
Moon *et al*./2013[24]	Korea (Apr. 2009-Jul. 2011)	2/244	0.8%	None	ND	ND	ND	ND
Kumar *et al*./2009[25]	India (Jul. 2004-Nov. 2007)	1/40 (Allo)	2.5% (Allo)	ND	ND	ND	0/1	1
Ullah *et al*./2007[26]	Pakistan (Jul. 2001-Sep. 2006)	4/154 (Allo)	2.6% (Allo)	Done	ND	ND	0/1	1
Al-Anazi *et al*./2007[27]	Saudi Arabia (1991–2002)	3/103	2.9%	Partially done	ND	1 to 12 m	2/1	ND
Park *et al*./2006[28]	Korea (2001–2002)	8/379	2.1%	None	ND	ND	ND	2
Garces-Ambrossi *et al*./2005[29]	USA (1993–2001)	4/577 (Allo)	0.7% (Allo)	ND	0.01–0.03%	60 to 300 d	3/1	ND
Ahmed *et al*./2005[30]	Pakistan (Jul. 2001- Oct. 2003)	4/50 (Allo)	8% (Allo)	Partially done	ND	42 to 525 d	1/3	ND
Yoo *et al*./2004[31]	Korea (1998–1999)	8/242 (Allo)	3.3% (Allo)	None	ND	176 to 734 d	ND	0
Lee *et al*./2004[32]	Korea (Feb. 1996 -Jul.2003)	7/156 (Allo) 2/139 (Auto)	4.5% (Allo) 1.4% (Auto)	Partially done	ND	30 to 165 d	7 /2	0
George *et al*./2004[33]	India (1986–2001)	9/304 (Allo)	3.0% (Allo)	ND	ND	ND	1/8	0
Erdstein *et al*./2004[34]	Australia (1999–2001)	4/127 (Allo)	3.1% (Allo)	ND	ND	3.3 to 15 m	3/1	2
Ku *et al*./2001[5]	Taiwan (Mar.1994-Mar. 2000)	8/255 (Allo) 0/95 (Auto)^b^	3.1% (Allo) 0% (Auto)	ND	13.1 times greater than general population	1 to 33.5 m	8/0	1
de la Camara *et al*./2000[6]	Spain (1976–1998)	12/2866 (Allo) 8/5147 (Auto)	0.4% (Allo) 0.2% (Auto)	Partially done	2.21 times greater than general population	11 to 3337 d	15/5	3
Budak-Alpdogan *et al*./2000[35]	Turkey (Jan. 1988-Aug. 1998)	5/351 (Allo)	1.4% (Allo)	77 (21.9%)	35.4 per 100 000 PY	10 to 47 m	4/1	ND
Aljurf *et al*./1999[36]	Saudi Arabia (1986–1997)	4/641 (Allo)	0.6% (Allo)	ND	ND	120 to 600 d	2/2	2
Ip *et al*./1998[10]	Hong Kong (1991–1994)	10/183^b^	5.5%	5 (2.7%)	ND	23 to 550 d	10	ND
Roy *et al*./1997[37]	USA (1974–1994)	1/1486(Allo) 0/755 (Auto)	0.1% (Allo) 0% (Auto)	ND	0.01%	7 to 100 d	0/1	ND
Lee *et al*./(this study)	Korea (2004–2011)	21/824 (Allo)	2.5% (Allo)	Partially done	9.1 times greater than general population	49 to 886 d	11/10	1

Allo, allogeneic; Auto, autologous; HSCT, hematopoietic stem cell transplantation; INH, isoniazid; ND, Not described; PY, patient-year; TB, tuberculosis.

^a^ Of 211 cases in a non-immunocompromised control group, the TB incidence was 0%.

^b^ Only pulmonary tuberculosis was included.

The incidence of TB following HSCT is found to be 10–40 times higher than that of the general population [2]. The cumulative incidence of TB disease in our study was 9.1 times higher than that of the general population in Korea. In Taiwan, pulmonary TB in allogeneic HSCT was 13.1-fold higher [5]. In Spain, de la Camara *et al*. reported that allogeneic HSCT recipients showed 2.95 times higher relative risk of TB incidence compared with the general population [6]. It seems that geographic location and follow-up duration may influence the differences in the incidence of TB disease.

Several risk factors for the development of TB after HSCT have been reported, including AML, CML, MDS, the use of busulfan, cyclophosphamide, TBI, corticosteroid therapy, mismatched allografts, GVHD, or history of previous TB infection [2]. Our study shows that extensive chronic GVHD is associated with the development of TB. Donor type, underlying hematological disease, conditioning regimen, and acute GVHD were not risk factors according to our study. A TBI-based conditioning regimen was given to >70% of the recipients in our center, which seems to affect the result. The other center in Korea previously reported that HSCT patients with TBI-based conditioning are likely to have TB disease, while TBI-based conditioning was given to 45% of patients with HSCT [32, 38].

The extrapulmonary TB rate tends to be higher than that of the general population since decreased cell-mediated immune responses cannot restrict TB infection [10]. The rate of extrapulmonary TB is reportedly 15–20% in the Korean general population [39]. Our result was also much higher (47.6%) than that in the general population. Table 5 shows a diverse range of extrapulmonary TB rates. The reasons behind the wide range of extrapulmonary TB rates are due to various degrees of practices of close examinations, a high index of suspicion, and anti-TB treatment timing [10].

In this study, there was no association with the IGRA results and progression of active TB disease, which corresponds well with those found in the earlier studies. Sester *et al*. also reported that the IGRA or tuberculin skin test poorly predicted the progression of active TB in immunocompromised hosts [23]. The immunosuppression state of HSCT recipients would influence IGRA test accuracy [40].

INH prophylaxis has been used to prevent active TB in LTBI patients [2]. However, INH prophylaxis has several problems, including hepatotoxicity, drug interaction with immunosuppressants, and manifestation of resistance to INH [2, 41, 42]. Therefore, the efficacy of INH prophylaxis in HSCT recipients needs to be determined. The result of the present study is that INH prophylaxis did not significantly alter the incidence of TB disease, which is similar to the results of earlier studies [32, 41]. However, these findings differ from suggestions of other reports, which recommend INH prophylaxis for HSCT recipients [3, 30]. Despite the long follow-up period, here we reported TB disease in 21 patients in the whole group and only five patients in group B (INH prophylaxis group) due to TB being a relatively rare disease. The small number of patients may not be sufficient for detecting significant differences for evaluating the effect of INH prophylaxis. However, among the 21 TB patients, only one took INH prophylaxis for 3 months due to poor compliance. Well-designed prospective clinical trials are needed to estimate the efficacy of INH prophylaxis. Also, the ideal INH prophylaxis duration is debatable because TB disease usually occurs in the late post-HSCT period [31, 35]. In our report, patients developed TB at a median of 1 year after HSCT.

One recent study reported that Korea shows a low rate of multidrug-resistant TB (<2000 cases in 2011) [43]. In 2004, the proportion of INH resistance among the total number of notified new TB cases was 9.9% in Korea [44]. Our study population showed only one patient with INH resistance among 6 antimicrobial susceptibility test-proven TB cases, and this patient improved when underwent second-line anti-TB treatment. However, Korea is one of the countries in which multidrug-resistant TB has been increasing [45]. Therefore, when patients with TB are treated, resistance to anti-TB medications needs to be taken into consideration. Also, the value of INH prophylaxis needs to be assessed carefully.

The present study has some limitations. First, it was retrospective, which caused some problems in that specific information about the history of previous TB infection and data of therapeutic approach of chronic GVHD in patients without current TB disease were missing from the medical records. Second, it included a small number of patients with TB. Third, in group B, INH prophylaxis was not given to all IGRA positive patients. Regardless of these limitations, major strengths of our study include a long follow-up period (11 years) and the study population, a relatively homologous large number of allogeneic HSCT patients with only AML, CML, MDS, or MM. Also, the cumulative incidence calculated by Gray’s test considered the competing risks and we used a standardized incidence ratio when making comparisons with the general population.

In conclusion, our study provides a useful evaluation for allogeneic HSCT patient group who is at high risk of contracting TB disease, compared to the general population. TB should be considered when evaluating allogeneic HSCT patients, especially those with extensive chronic GVHD. INH prophylaxis did not statistically change the incidence of TB; however, there is a tendency of decreased TB incidence among the INH prophylaxis group. More research is warranted on the efficacy of INH prophylaxis in allogeneic HSCT recipients in an intermediate TB burden country.

## Supporting information

S1 Data Minimal data set for tuberculosis in allogeneic hematopoietic stem cell transplantation recipients. (PDF)
