# Supplementary material for: The demanding attention of tuberculosis in allogeneic hematopoietic stem cell transplantation recipients: High incidence compared with general population
Source: PLoS One. 2017 Mar 9;12(3):e0173250. doi: 10.1371/journal.pone.0173250 (PMC5344370; doi:10.1371/journal.pone.0173250)
Supplement: S1 Data — (PDF) [file pone.0173250.s001.pdf]

Minimal data set for tuberculosis in allogeneic hematopoietic stem cell transplantation recipients

Serial No : **Name :**

Age/Sex : **/** (age at HSCT) **Chart No :**

Underlying Disease :

□ AML □ ALL □ CML

□ MDS □ MM □ Others ( )

BMT date :

Type of SCT

□ Syngeneic □ Matched-related □ Matched-unrelated □ Family mismatch

Stem cell Source

□ BM □ PBS □ BM + PBSC □ Cord blood

Conditioning regimen (TBI) □ TBI based □ No-TBI based

Conditioning regimen (ATG) □ ATG □ No-ATG

Conditioning Intensity □ Myeloablative □ Non-myeloablative (mini-)

GVHD prophylaxis

□ Cyclosporine □ Methotrexate □ FK506 □ MMF □ Others ( )

Acute GVHD □ None □ Yes

Overall grade :

aGVHD date :

Involved organ :

Outcome □ Improvement □ Aggravation

Chronic GVHD □ None □ Limited □ Extensive

cGVHD date :

Involved organ :

Grade □ Mild □ Moderate □ Severe

Outcome □ Improvement □ Aggravation

Relapse of hematologic disease □ Yes □ No

Relapse date :

Death □ Yes □ No

Date of death :

Cause of death :

Last follow up date :

IGRA □ Positive □ Negative □ Not done

INH prophylaxis □ Yes □ No □ Not done

If yes, duration :

If no, the reason :

Events during INH prophylaxis

□ Drug toxicity ( )

□ Relapse of hematologic disease ( )

□ Aggravation of GVHD ( )

□ Others ( )

TB after HSCT □ Yes □ No

Date of TB Diagnosis :

Dx of tuberculosis

□ **PROVEN** □ Culture, culture site :

□ **PROBABLE** □ AFB Smear, site :

□ Tissue histology, site :

□ Fluid cytology, site :

□ **POSSIBLE** □ Radiologic result, site : □ Improve after anti TB medication

□ Not improve after antibacterial and antifungal medication

Past Hx of TB □ Yes □ No

Sx & Sign

□ Fever (duration :) □ Cough (duration :)

□ Sputum (duration :) □ Weight loss (duration :)

□ Night sweat (duration :) □ Dyspnea (duration :)

□ Chest pain (duration :) □ Others ( )

Site of Infection

□ Pulmonary

□ Extrapulmonary (Pleura, Liver, Spleen, BM, CNS, L/N, Others ( ))

□ Disseminated (involved organs :)

Anti TB medication

Duration of Medication ( ) months

Regimen □ INH □ RFP □ EMB □ PZ □ others ( )

Change to 2nd line drugs? □ Yes □ No

If yes, the reasons : the regimen : duration :

Response to anti-TB medication

□ Cure of TB □ Improvement of TB □ Aggravation of TB □ Death due to TB

Events during anti-TB medication

□ IRIS, site :

□ Drug toxicity ( )

□ Others ( )

Results of susceptibility test (Senstive, Resistant)

INH (S, R), RFP (S, R) MBT (S, R), PZ (S, R),

OFX (S, R), CS (S, R), SM (S, R), PAS (S, R),

Prithionamide (S, R), Enviomycin (Tuberlactinomycin) (S, R),

Others ( ) (S, R), Others ( ) (S, R)
